# The critical role of the linear plasmid lp36 in the infectious cycle of *Borrelia burgdorferi*

**DOI:** 10.1111/j.1365-2958.2007.05746.x

**Published:** 2007-06-01

**Authors:** Mollie W Jewett, Kevin Lawrence, Aaron C Bestor, Kit Tilly, Dorothee Grimm, Pamela Shaw, Mark VanRaden, Frank Gherardini, Patricia A Rosa

**Affiliations:** 1 Laboratory of Zoonotic Pathogens, Rocky Mountain Laboratories, National Institute of Allergy and Infectious Diseases, National Institutes of Health Hamilton, MT 59840, USA.; 2 Biostatistics Research Branch, National Institute of Allergy and Infectious Diseases, National Institutes of Health Bethesda, MD 20892, USA.

## Abstract

*Borrelia burgdorferi*, the aetiological agent of Lyme disease, follows a life cycle that involves passage between the tick vector and the mammalian host. To investigate the role of the 36 kb linear plasmid, lp36 (also designated the *B. burgdorferi* K plasmid), in the infectious cycle of *B. burgdorferi*, we examined a clone lacking this plasmid, but containing all other plasmids known to be required for infectivity. Our results indicated that lp36 was not required for spirochete survival in the tick, but the clone lacking lp36 demonstrated low infectivity in the mammal. Restoration of lp36 to the mutant strain confirmed that the infectivity defect was due to loss of lp36. Moreover, spirochetes lacking lp36 exhibited a nearly 4-log increase in ID_50_ relative to the isogenic lp36^+^ clone. The infectivity defect of lp36-minus spirochetes was localized, in part, to loss of the *bbk17* (*adeC*) gene, which encodes an adenine deaminase. This work establishes a vital role for lp36 in the infectious cycle of *B. burgdorferi* and identifies the *bbk17* gene as a component of this plasmid that contributes to mammalian infectivity.

## Introduction

*Borrelia burgdorferi*, the aetiological agent of Lyme disease, has an enzootic life cycle that requires persistence in two disparate environments, the arthropod vector and the mammalian host (Burgdorfer *et al*., 1982; Donahue *et al*., 1987; Lane *et al*., 1991; Lane, 1994). In the wild, *B. burgdorferi* spirochetes cycle between small mammals, such as the white-footed mouse, *Peromyscus leucopus*, and *Ixodes* sp. ticks (Burgdorfer *et al*., 1982; Donahue *et al*., 1987; Lane *et al*., 1991; Lane, 1994). Humans, although not reservoirs for *B. burgdorferi*, may become infected when fed upon by infected ticks. Survival in nature of *B. burgdorferi*, an obligate parasite, entails: (i) infection of and persistence in the tick (ii) transmission from tick to mammal during a blood meal (iii) persistent infection in the mammal and (iv) reacquisition from the mammal by feeding ticks (Steere *et al*., 1983; Steere, 2001). The development of tools for genetic manipulation of *B. burgdorferi* has facilitated the identification of molecular determinants required for the survival of this spirochete throughout its life cycle (Rosa *et al*., 2005).

*Borrelia burgdorferi* harbours a segmented genome that includes a small, 900 kb linear chromosome and as many as 23 circular and linear plasmids, ranging in size from 5 kb to 56 kb (Fraser *et al*., 1997; Stevenson *et al*., 1997; 1998; Casjens *et al*., 2000; Miller *et al*., 2000). Genome sequence analyses of *B. burgdorferi* type strain B31 reveal that a majority of the plasmid-encoded open reading frames have no database matches (Fraser *et al*., 1997; Casjens *et al*., 2000). The *B. burgdorferi* genome is unstable during *in vitro* passage and many of the plasmids can be lost during this process. Loss of certain plasmids is tightly correlated with loss of infectivity and persistence in mice and ticks (Schwan *et al*., 1988a; Norris *et al*., 1995; Xu *et al*., 1996; 2005; Purser and Norris, 2000; Labandeira-Rey and Skare, 2001; McDowell *et al*., 2001; Labandeira-Rey *et al*., 2003; Grimm *et al*., 2004; 2005; Lawrenz *et al*., 2004; Strother *et al*., 2005), while not affecting growth *in vitro*.

For example, the linear plasmid (lp) lp25 is critical for infection of the mouse and tick (Purser and Norris, 2000; Labandeira-Rey and Skare, 2001; Labandeira-Rey *et al*., 2003; Grimm *et al*., 2004; 2005; Revel *et al*., 2005; Strother *et al*., 2005) and linear plasmid lp28-1 is necessary for persistence in the mouse (Zhang *et al*., 1997; Purser and Norris, 2000; Labandeira-Rey and Skare, 2001; Labandeira-Rey *et al*., 2003; Grimm *et al*., 2004; Revel *et al*., 2005; Xu *et al*., 2005). At least some of the genetic elements present on lp25 that contribute to *in vivo* survival have been identified. The *pncA* (*bbe22*) gene on lp25 encodes a nicotinamidase, an enzyme that is sufficient to restore infectivity in mice and ticks to clones lacking the entire lp25 plasmid (Purser *et al*., 2003; Grimm *et al*., 2005; Strother *et al*., 2005). In addition, lp25 harbours the *bptA* (*bbe16*) gene that encodes a putative lipoprotein that is important for persistence of *B. burgdorferi* in the tick (Revel *et al*., 2005). The lp28-1 plasmid carries the *vlsE* locus, which undergoes antigenic variation during mammalian infection and is presumed to be required for evasion of the host immune system and establishment of a persistent infection (Zhang *et al*., 1997; Zhang and Norris, 1998; Eicken *et al*., 2001; Indest *et al*., 2001; McDowell *et al*., 2002; Ohnishi *et al*., 2003).

Unlike lp25 and lp28-1, the lp36 plasmid has rarely been observed to be lost by B31 *B. burgdorferi* during *in vitro* passage (Purser and Norris, 2000) and its potential role in the *B. burgdorferi* infectious cycle has not been examined. We have identified a low passage B31 clone that has lost lp36, but harbours all other plasmids known to be important for virulence, thereby allowing investigation of the role of this plasmid in the *B. burgdorferi* infectious cycle. The lp36 plasmid of strain B31 is a linear plasmid of approximately 36 kb encoding 54 putative open reading frames, seven of which appear to be pseudogenes (Fraser *et al*., 1997; Casjens *et al*., 2000). Moreover, a majority of the genes found on lp36 from strain B31 have no predicted function (Casjens *et al*., 2000). Of the few genes on lp36 that have a putative function (Casjens *et al*., 2000), *bbk32 has* been shown to encode a fibronectin binding protein (Probert and Johnson, 1998; Parveen and Leong, 2000; Probert *et al*., 2001) that may be involved in *B. burgdorferi* mammalian infectivity (Li *et al*., 2006; Seshu *et al*., 2006). We found that spirochetes lacking lp36 did not readily survive in the mammal but displayed no deficiency in the tick. Mouse infection was restored by reconstitution of the lp36 plasmid in the mutant clone, demonstrating that the infectivity defect resulted from the loss of lp36. Furthermore, we established that the *bbk17* gene of lp36 encodes an adenine deaminase (AdeC) and is a genetic component on lp36 that contributes to mammalian infectivity and is sufficient to restore mouse infectivity to spirochetes lacking lp36. Our work establishes a critical role for the lp36 plasmid in the *B. burgdorferi* infectious cycle.

## Results

### *Borrelia burgdorferi* lacking lp36 are unable to establish infection in a mouse by needle inoculation

**Fig. 1:**
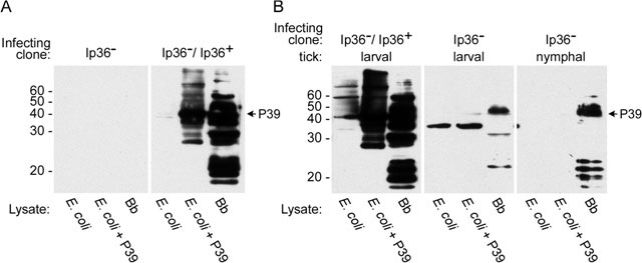
Serological responses of mice infected with various clones of *B. burgdorferi* A. Immunoblot analysis of sera collected 6 weeks post inoculation from C3H/HeN mice injected with a target dose of 5 × 10^3^ spirochetes of *B. burgdorferi* clones lacking or containing lp36. Protein lysates tested for seroreactivity include: *E. coli*, lysate of *E. coli* carrying a cloning vector; *E. coli* + P39, lysate of *E. coli* carrying a cloning vector producing the *B. burgdorferi* P39 protein; Bb, lysate of *B. burgdorferi*. Representative results with the serum of mice inoculated with each *B. burgdorferi* clone are shown. The position of the P39 protein is indicated with an arrow to the right of the panel. The positions of markers to the left of the panel depict molecular weights in kilodaltons. B. Immunoblot analysis of sera collected 7 weeks post tick feeding from RML mice fed on by either approximately 100 *I. scapularis* larvae or approximately 20 *I. scapularis* nymphs infected with *B. burgdorferi* clones containing or lacking lp36. Protein lysates tested were as described for A. The first panel shows representative results obtained with the serum of mice fed on by larval or nymphal ticks infected with spirochetes containing lp36. The second panel shows an example of results obtained with the serum from the two seropositive mice fed on by larval ticks infected with spirochetes lacking lp36. The third panel shows an example of results obtained with the serum from the two seropositive mice fed on by nymphal ticks infected with spirochetes lacking lp36. The position of the P39 protein is indicated with an arrow to the right of the panel. The positions of markers to the left of the panel depict molecular weights in kilodaltons. Comparable serum dilutions and exposure times were used for all blots.

To evaluate the ability of *B. burgdorferi* lacking lp36 to adapt to and survive in the mammalian environment, we took advantage of a derivative of the low passage clone A3-M9 (Table 1) that lacks this plasmid, but contains all other plasmids known to be required for infectivity (Table 1). For direct experimental comparison, we reintroduced lp36 marked with a gentamicin resistance cassette into the lp36-minus clone in order to obtain an isogenic lp36^+^ clone (A3-M9 lp36-minus/lp36-gent) (Table 1). C3H/HeN mice were inoculated at a target dose of 5 × 10^3^ spirochetes by a combination of intraperitoneal and subcutaneous routes with A3-M9 lp36-minus or the isogenic clone to which the lp36 plasmid was restored (A3-M9 lp36-minus/lp36-gent) (Table 2). Four weeks post inoculation, the mice were bled and their sera were assessed for reactivity with *B. burgdorferi* antigens. None of the six mice inoculated with lp36-minus spirochetes showed evidence of seroconversion, in contrast to the 14 out of 15 mice that became infected with lp36^+^ spirochetes (Table 2, Fig. 1A). Moreover, no lp36-minus spirochetes were isolated from any of the mouse tissues examined (Table 2). Lp36^+^ spirochetes were reisolated from all three tissue sources of seropositive mice (Table 2). *In vitro* growth studies confirmed that lp36-minus spirochetes had no general growth defect relative to lp36^+^ spirochetes. Both clones demonstrated a doubling time of approximately 5.4 h and reached a maximum stationary phase density of 4.0 × 10^8^ spirochetes ml^−1^, eliminating the possibility that *in vivo* phenotypic differences were a consequence of *in vitro* growth differences between the two clones. These data provided the first direct evidence of a critical role for the lp36 plasmid in the ability of *B. burgdorferi* to colonize and establish a persistent infection in a mouse.

**Table 1:** *B. burgdorferi* clones used in this study.

Clone	Introduced DNA	Plasmid(s) missing	Reference
A3-M9	None	cp9, lp21	Tilly *et al*. (2004)
A3-M9 lp36-minus	None	cp9, lp21, lp36	This work
A3/lp36-gent	lp36 + *flaB*_p_-*aacC1*	cp9	This work
A3-M9 lp36-minus/lp36-gent	lp36 + *flaB*_p_-*aacC1*	cp9, lp21	This work
A3-M9 lp36-minus/pBSV2G	pBSV2G	cp9, lp21, lp36	This work
A3-M9 lp36-minus/pBSV2G *bbk17*	pBSV2G *bbk17*	cp9, lp21, lp36	This work
A3-M9 Δ*bbk17*::*flgB*_*p*_*-kan*/pBSV2G	Δ*bbk17*::*flgB*_p_-*kan*, pBSV2G	cp9, lp21	This work
A3-M9 Δ*bbk17*::*flgB*_p_-*kan*/pBSV2G *bbk17*	Δ*bbk17*::*flgB*_p_-*kan*, pBSV2G *bbk17*	cp9, lp21	This work

**Table 2:** Infection of mice with *B. burgdorferi* clones lacking or containing lp36.^a^

		Reisolation from tissues^d^				
				
Clone	Serology^c^	Ear	Bladder	Joint	No. of mice infected/total	*P*-value^e^
A3-M9 lp36-minus^b^	0/6	0/6	0/6	0/6	0/6	0.0001
A3-M9 lp36-minus/lp36-gent^f^	14/15	14/15	14/15	14/15	14/15	

a Mice were infected by needle inoculation with a target dose of 5 × 10^3^ spirochetes as described in the *Experimental procedures*.

b Pooled data from two separate infection experiments.

c Assessed by immunoblot analysis with cell lysates of *B. burgdorferi* and *E. coli* producing P39 recombinant protein at 3 and 6 weeks post inoculation.

d Mice were sacrificed and tissues harvested 6 weeks post inoculation.

e The *P*-value was calculated by Fischer's exact test for the number of infected mice in the two groups.

f Pooled data from three separate infection experiments.

### The lp36 plasmid is not required for artificial infection of, or survival within, *Ixodes scapularis* ticks

**Fig. 2:**
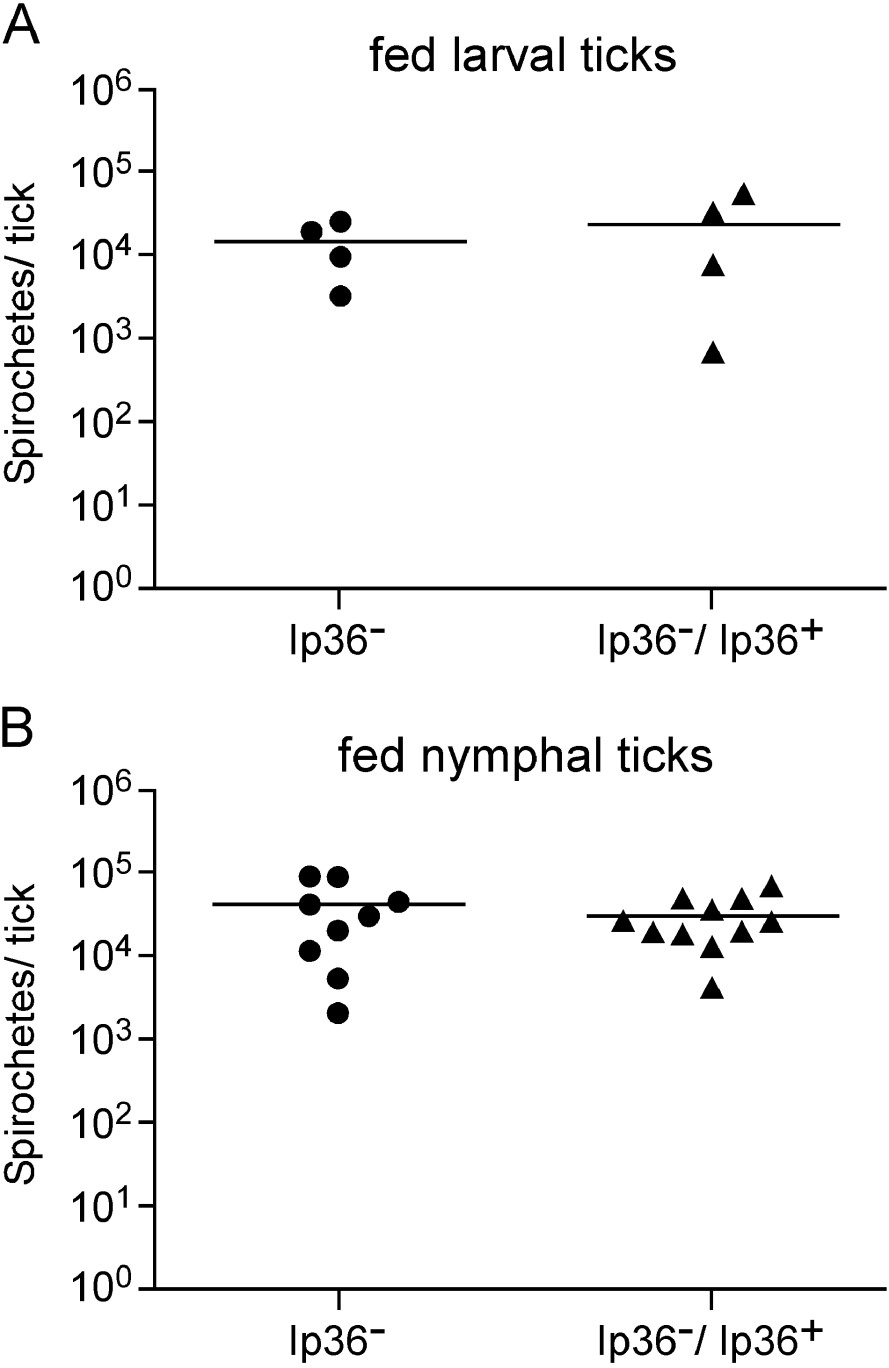
Colonization of tick midguts by various *B. burgdorferi* clones lacking or containing lp36 A. *I. scapularis* larvae were artificially infected by immersion in liquid cultures of *B. burgdorferi* clones and then fed to repletion on naive mice. The number of spirochetes per larval tick was quantified by plating of dilutions of triturated whole fed larvae in solid BSK medium and determining colony-forming units. Spirochete densities were determined 7 days post larval feeding to repletion. Symbols represent the number of spirochetes per individual fed larval tick. B. Following the molt, nymphs were fed on uninfected mice and the number of spirochetes per nymph was determined 7 and 14 days post feeding to repletion as described in A. Symbols represent the number of spirochetes per individual fed nymphal tick.

In addition to survival in the mammalian host, the *B. burgdorferi* infectious cycle requires colonization and persistent infection of the tick vector. Because spirochetes lacking lp36 did not establish a mouse infection by needle inoculation, we were unable to naturally infect ticks with lp36-minus *B. burgdorferi* by directly feeding them on infected mice. In lieu of this, cohorts of approximately 100 *I. scapularis* larvae were artificially infected with clone A3-M9 lp36-minus or A3-M9 lp36-minus/lp36-gent by immersion in borrelial cultures (Policastro and Schwan, 2003; Grimm *et al*., 2005). Seven days after feeding to repletion on mice, larvae were assessed for the presence and abundance of spirochetes in their midguts by immunofluorescence assay (IFA) and plating of dilutions of triturated whole ticks in solid BSK medium. The *B. burgdorferi* clones lacking or containing lp36 demonstrated equal ability to infect larval ticks (10/10 and 16/16 respectively). Furthermore, no difference in the average number of spirochetes per larval tick was detected between the clones following the blood meal (Fig. 2A, *P* > 0.05).

A similar proportion of ticks infected with the lp36-minus or lp36^+^*B. burgdorferi* clones (22/29 and 22/27 respectively) retained spirochetes through the molt to nymphs, indicating that spirochetes lacking lp36 do not demonstrate reduced fitness compared to lp36^+^ spirochetes. Ticks that maintained *Borrelia* infections with either clone through the molt harboured comparable spirochete densities following the nymphal blood meal (Fig. 2B, *P* > 0.05). These results indicate that the lp36 plasmid is not required by *B. burgdorferi* for colonization of larval ticks, replication of spirochetes within fed larvae, transstadial persistence, or replication of spirochetes within nymphs after tick feeding. Together our data suggest that lp36 is required for *B. burgdorferi* survival in the mammalian environment, but not in the tick environment.

### Spirochetes lacking lp36 are attenuated for mouse infection by tick bite

The lp36 plasmid had no effect on tick infection and equal spirochete loads were detected in infected ticks following the blood meal regardless of the presence or absence of lp36 (Fig. 2). Therefore, we tested whether or not the mice became infected with lp36-minus *B. burgdorferi* when fed upon by infected ticks, thus representing the natural route of mammalian infection. Unlike mice inoculated by needle, RML mice fed on by larval ticks artificially infected with *B. burgdorferi* clone A3-M9 lp36-minus demonstrated reduced, but detectable levels of infection (Table 3). Two out of the five mice tested in three separate experiments developed weak seroreactivity towards *Borrelia* antigens (Fig. 1B, Table 3) and reisolation of lp36-minus spirochetes was limited to the ear tissue of these mice (Table 3). The absence of the lp36 plasmid in the ear reisolates was confirmed by Southern blot analysis (data not shown). In contrast to the lp36-minus clone and consistent with previous results by needle inoculation, four out of five of the mice fed on by larval ticks artificially infected with *B. burgdorferi* clone A3-M9 lp36-minus/lp36-gent became infected (Table 3). Of those seropositive mice, lp36^+^ spirochetes were reisolated from all tissues examined (Table 3). Similar numbers of spirochetes were detected in all larval ticks regardless of whether or not the mice upon which they had fed became infected, suggesting there was no difference in the inoculum received by each mouse (Fig. 2 and data not shown).

**Table 3:** Tick bite infection of mice with *B. burgdorferi* clones lacking or containing lp36.

	Mouse infection by larval tick bite^a^						Mouse infection by nymphal tick bite^a^					
		
		Reisolation from tissues^c^						Reisolation from tissues^c^				
								
Clone	Serology^b^	Ear	Bladder	Joint	No. mice infected/total	*P*-value^d^	Serology^b^	Ear	Bladder	Joint	No. mice infected/total	*P*-value^d^
A3-M9 lp36-minus	2/5	2/5	0/5	0/5	2/5	0.52	2/5	2/5	1/5	0/5	2/5	0.17
A3-M9 lp36-minus/lp36-gent	4/5	4/5	4/5	4/5	4/5		4/4^e^	4/4	4/4	4/4	4/4	

a Number of mice infected/number of mice analysed. Pooled data from three separate tick feeding experiments unless otherwise noted.

b Assessed by immunoblot analysis with cell lysates of *B. burgdorferi* and *E. coli* producing P39 recombinant protein at 3, 5 and 7 weeks post inoculation.

c Mice were sacrificed and tissues harvested 7 weeks post inoculation.

d The *P*-value was calculated by Fischer's exact test for the number of infected mice in the two groups.

e Pooled data from two separate tick feeding experiments.

Similar results were obtained after feeding infected nymphs on mice. Weak seroreactivity towards *Borrelia* antigens was detected in two out of the five mice fed upon by lp36-minus-infected nymphs (Fig. 1B, Table 3). Lp36-minus spirochetes were reisolated from the ear tissue and the ear and bladder tissues of the two weakly seropositive mice, respectively (Table 3). All of the four mice fed on by nymphs infected with lp36^+^ spirochetes demonstrated robust seroconversion and spirochetes were reisolated from all three tissue sources (Table 3). As for larval ticks, similar numbers of spirochetes were detected in all nymphal ticks regardless of whether or not the mice upon which they had fed became infected, suggesting there was no difference in the inoculum received by each mouse (Fig. 2 and data not shown).

**Fig. 3:**
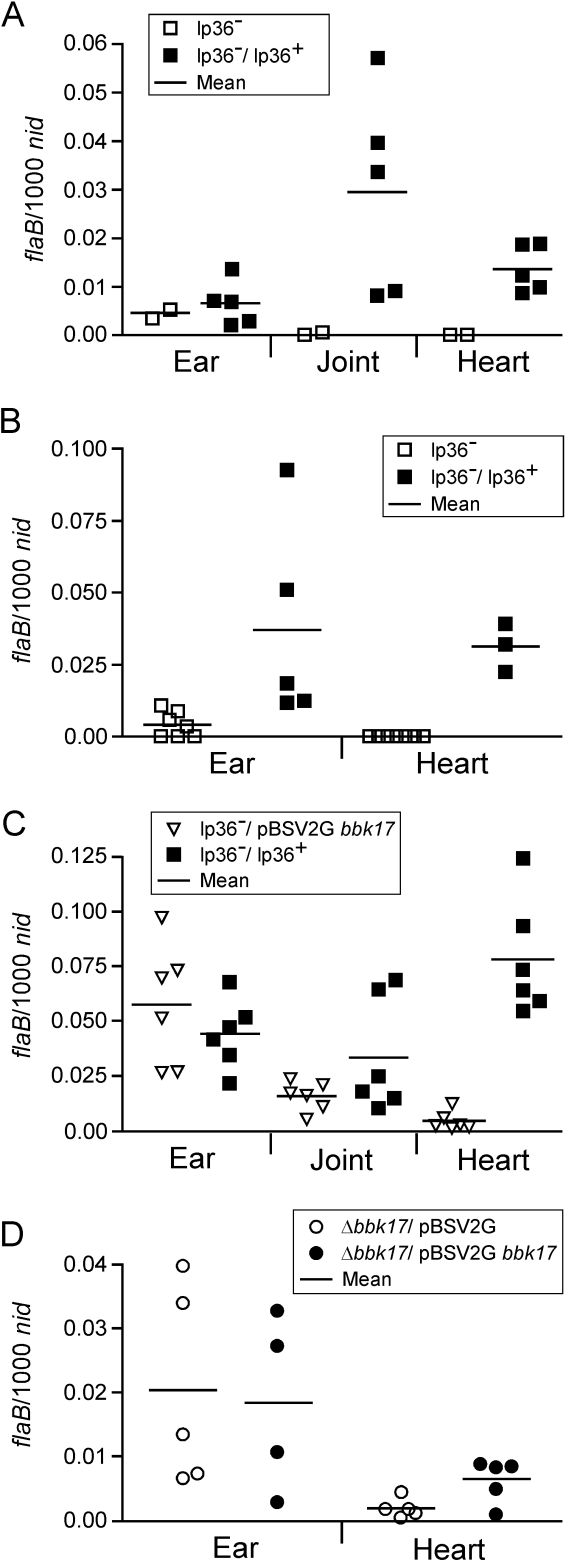
Quantitative assessment of spirochetal loads in infected mouse tissues A. DNA was isolated from ear, heart and joint tissues from a subset of RML mice that were reisolation-positive 7 weeks post feeding by larval or nymphal ticks infected with lp36-minus (□ lp36^-^) or lp36-minus/lp36-gent (▪ lp36–/lp36+) spirochetes. Samples were assessed for spirochete *flaB* and murine nidogen DNA copies by qPCR. The data are expressed as *flaB* copies per 1000 nidogen copies. Each data point represents the average of triplicate measures from the tissue DNA of an individual mouse. B. DNA was isolated from ear and heart tissues (joint tissue was not available from these experiments) from C3H/HeN mice that were reisolation-positive 4 weeks post inoculation with 1 × 10^7^ or 1 × 10^8^ lp36-minus (□ lp36^-^) or lp36-minus/lp36-gent (▪ lp36–/lp36+) spirochetes. Data shown are pooled data from individual mice inoculated with either 1 × 10^7^ or 1 × 10^8^ spirochetes. The data were collected and presented as described in A. C. DNA was isolated from ear, joint and heart tissues from C3H/HeN mice that were reisolation positive 6 weeks post inoculation with 5 × 10^3^ lp36-minus/pBSV2G *bbk17* (∇ lp36^-^/pBSV2G *bbk17*) or lp36-minus/lp36-gent (▪ lp36–/lp36+) spirochetes. The data were collected and presented as described in A. D. DNA was isolated from ear and heart tissues (joint tissue was not available from these experiments) from C3H/HeN mice that were reisolation-positive 4 weeks post inoculation with 1 × 10^4^Δ*bbk17*/pBSV2G (○) or Δ*bbk17*/pBSV2G *bbk17* (•) spirochetes. The data were collected and presented as described in A.

The spirochete loads in tissues were assessed by quantitative polymerase chain reaction (qPCR) of mice infected by tick bite with spirochetes either lacking or containing lp36. No difference in spirochete burden was detected in the ear tissue of mice infected with either clone (Fig. 3A). However, the joint and heart tissues from mice infected with lp36^+^ spirochetes harboured a statistically greater bacterial load compared with those mice infected with spirochetes lacking lp36 (Fig. 3A, *P* < 0.05). Furthermore, the numbers of spirochetes in the joint and heart tissues from mice infected with spirochetes lacking lp36 were not different from that of uninfected tissues (data not shown). These data were consistent with the observation that reisolation of lp36-minus spirochetes was mainly limited to the ear (Table 3).

Successful completion of the *B. burgdorferi* tick-mouse infectious cycle requires the acquisition of spirochetes by uninfected larval ticks while feeding on an infected mouse. Because infected ticks were able to transmit both lp36-minus and lp36^+^*B. burgdorferi* to a limited number of mice, we were now in a position to ask whether or not uninfected larvae could acquire these spirochetes naturally by feeding on infected mice, in order to complete the infectious cycle. Approximately 100 uninfected *I. scapularis* larvae were fed to repletion on mice infected with either *B. burgdorferi* clone A3-M9 lp36-minus or A3-M9 lp36-minus/lp36-gent. Larvae were assessed for the presence and density of spirochetes in the midguts 7 days post feeding by IFA and plating of dilutions of triturated whole ticks. No spirochetes were detected by either IFA or plating of nine larvae that fed on mice infected with spirochetes lacking lp36, while eight out of nine of the larvae that fed on mice infected with lp36^+^*B. burgdorferi* harboured live spirochetes.

There was no statistical difference in the absolute number of mice that became infected by tick bite with lp36-minus compared with lp36^+^*B. burgdorferi* (Table 3). Because there was evidence that the number of infected tissues was reduced in mice infected with spirochetes lacking lp36 compared with mice infected with lp36^+^ spirochetes, the data were also compared using an exact permutation test, which took into account the average number of infected tissues per mouse. The *P*-values for the difference between the number of infected tissues per mouse infected with lp36-minus or lp36-minus/lp36-gent spirochetes by larval or nymphal tick bite were 0.05 and 0.02 respectively. Although there was little to no lp36-dependent statistical difference in mouse infection by tick bite, there was a trend towards a difference that was not detectable given the small sample size. Moreover, there was a biological difference between the mice infected by tick bite with lp36-minus compared to lp36^+^ spirochetes. The serological response to *Borrelia* proteins of mice infected with lp36-minus spirochetes was reduced relative to mice infected with lp36^+^ spirochetes (Fig. 1B), lp36-minus spirochetes were reisolated from limited tissues (Table 3), and were only detected in the ear tissue by qPCR (Fig. 3A). Finally, although a proportion of the mice fed on by ticks infected with lp36-minus spirochetes became infected, uninfected larvae did not acquire lp36-minus spirochetes by feeding on these mice, demonstrating that this infection does not permit efficient maintenance of *B. burgdorferi* in the infectious cycle.

### *Borrelia burgdorferi* lacking lp36 are highly attenuated relative to isogenic lp36*^+^* spirochetes

Our results demonstrate that the lp36 plasmid is essential for mouse infection by needle inoculation at a target dose of 5 × 10^3^ spirochetes and important for wild-type levels of mouse infectivity by tick bite. As the number of spirochetes transmitted by an infected tick is undefined and not amenable to manipulation, we were interested in quantifying the difference in the infectious capabilities of *B. burgdorferi* lacking or carrying the lp36 plasmid by determination of the 50% infectious dose (ID_50_) of these clones by mouse needle inoculation.

Groups of C3H/HeN mice were challenged with 10-fold increasing doses of spirochetes. Four weeks post inoculation, mouse infection status was determined by reisolation of spirochetes from mouse tissues (Table S1) and serological analysis (data not shown), which correlated completely. Reisolation of lp36-minus spirochetes from infected mice was limited to the ear tissue (Table S1). Spirochetes were reisolated from all three tissues of mice infected with lp36^+^ bacteria (Table S1). Spirochetal load was assessed in the ear and heart tissues of mice infected with 1 × 10^7^ and 1 × 10^8^ lp36-minus or lp36^+^ spirochetes. These two inocula were chosen for analysis of the spirochete burden in infected mouse tissues because it was at these doses that the greatest number of mice became infected with spirochetes lacking lp36, although only two mice were inoculated with lp36^+^ spirochetes at each of these doses (Table S1). Spirochete burdens in the ear and heart tissues of mice infected with both 1 × 10^7^ and 1 × 10^8^ spirochetes were dependent on the presence of lp36 (Fig. 3B, *P* < 0.05). Furthermore, the numbers of spirochetes in the heart tissue from mice infected with spirochetes lacking lp36 were no different from that of uninfected tissues (data not shown).

**Fig. 4:**
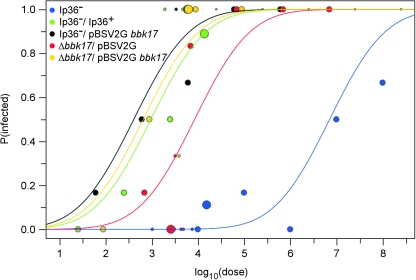
ID_50_ analysis of *B. burgdorferi* clones. Probit regression on adjusted spirochete doses was used to estimate the ID_50_ of *B. burgdorferi* clones. The plot represents the probability of infection versus the base 10 logarithm of the adjusted dose. Mouse infection data from both the ID_50_ and single dose experiments were included. Actual doses of the different inocula were adjusted for both the observed colony-forming units following plating on solid medium and the proportion of the *B. burgdorferi* population harbouring both lp25 and lp28-1. The point size is proportional to the number of mice inoculated at different target doses.

The dose–response curve for A3-M9 lp36-minus demonstrated a statistically significant right shift (*P* < 0.05) relative to the dose–response curve for A3-M9 lp36-minus/lp36-gent (Fig. 4). The ID_50_ for A3-M9 lp36-minus was estimated to be 7.1 × 10^6^[95% confidence interval (CI): 1.1 × 10^6^ − 5.8 × 10^7^] spirochetes compared with 9.5 × 10^2^ (95% CI: 1.5 × 10^2^ − 5.0 × 10^3^) spirochetes for A3-M9 lp36-minus/lp36-gent (Table S1). The ID_50_ value of 9.5 × 10^2^ for lp36^+^ spirochetes is similar to previously reported ID_50_ values for other B31-derived low passage, infectious *B. burgdorferi* clones (Tilly *et al*., 2006). Together these data indicate that *B. burgdorferi* lacking lp36 demonstrate an almost 4-log increase in ID_50_ relative to lp36^+^ spirochetes, further supporting the essential role of this plasmid in mammalian infectivity.

### The *bbk17* gene is a component of lp36 that contributes to mammalian infectivity

Our data have established that the lp36 plasmid is not required for infection and survival within the tick vector, but is critical for infection of the mammalian host. There are 56 putative open reading frames on lp36, most of which encode hypothetical proteins with no similarity to proteins of known function (Fraser *et al*., 1997; Casjens *et al*., 2000). Previous studies debate the *in vivo* importance of the lp36 gene *bbk32*, which encodes a fibronectin-binding protein (Probert and Johnson, 1998; Parveen and Leong, 2000; Probert *et al*., 2001). These earlier studies demonstrated either a single log increase in the infectious dose of the mutant compared with the complemented clone (1.8 × 10^3^ and 80 spirochetes respectively) (Seshu *et al*., 2006), or no difference in the ability of the mutant to infect and survive in the mouse at a dose of 10^5^ spirochetes (Li *et al*., 2006), suggesting that additional genes on lp36 are required for mammalian infectivity. The lp36 open reading frame BBK17 encodes a putative adenine deaminase, an enzyme involved in the conversion of adenine to hypoxanthine that is important for purine salvage in numerous bacteria and lower eukaryotes (Nygaard *et al*., 1996). Given the significance of this enzyme for microbial metabolism of purine nucleotides and nucleosides, and the apparent inability of *B. burgdorferi* to synthesize purines *de novo* (Fraser *et al*., 1997; Casjens *et al*., 2000), we hypothesized that the *bbk17* gene might contribute to the survival of *B. burgdorferi* in the mammalian environment.

To examine the ability of the *bbk17* gene alone to restore infectivity to spirochetes lacking the lp36 plasmid, clone A3-M9 lp36-minus was transformed with either a *Borrelia* shuttle vector harbouring the *bbk17* gene (pBSV2G *bbk17*) or pBSV2G alone. Infectious phenotypes of the resulting isogenic clones were examined by a combination of intraperitoneal and subcutaneous needle inoculation of a target dose of 5 × 10^3^ spirochetes into a total of 15 C3H/HeN mice in three separate experiments (Table 4). As previously established, spirochetes lacking the entire lp36 plasmid were severely impaired in their ability to survive in the mammalian host, because only one out of 15 mice was infected with clone A3-M9 lp36-minus/pBSV2G (Table 4). Strikingly, all 15 mice inoculated with lp36-minus spirochetes harbouring pBSV2G *bbk17* became infected, as determined by a strong antibody response to *Borrelia* antigens and reisolation of spirochetes from all three mouse tissues (Table 4). ID_50_ analysis confirmed the ability of the *bbk17* gene to restore wild-type infectivity to spirochetes lacking lp36. The dose–response curve for clone A3-M9 lp36-minus/pBSV2G *bbk17* was not statistically different from that of lp36^+^ spirochetes (*P* > 0.05) (Fig. 4). The ID_50_ for this clone was estimated to be 4.1 × 10^2^ (95% CI: 5.5 × 10^1^ − 2.1 × 10^3^) spirochetes (Table S1), which was similar to that determined for spirochetes harbouring the lp36 plasmid and 4 logs less than the ID_50_ for spirochetes lacking lp36 (Table S1). Because reintroduction of *bbk17* to *B. burgdorferi* lacking lp36 restored the ID_50_ to that of lp36^+^ spirochetes, the spirochetal load in tissues was assessed to determine whether reintroduction of the *bbk17* gene alone to *B. burgdorferi* lacking lp36 resulted in a spirochete burden comparable to mice infected with lp36^+^*B. burgdorferi*. Similar spirochetal loads were detected in the ear and joint tissues of mice infected with lp36-minus/pBSV2G *bbk17* and lp36^+^ spirochetes (Fig. 3C, *P* > 0.05). However, a statistically greater number of bacteria were present in the heart tissue of mice infected with lp36^+^ spirochetes compared with those harbouring the *bbk17* gene alone (Fig. 3C, *P* < 0.001). These data suggest that although reintroduction of the *bbk17* gene alone appears to be sufficient to restore mammalian infectivity to spirochetes lacking the entire lp36 plasmid, as measured by serology, spirochete reisolation from tissues and ID_50_, these spirochetes may be impaired for dissemination to deeper tissues such as the heart. Dissemination of this clone may be limited due to the absence of additional genes on lp36 (Terekhova *et al*., 2006), such as *bbk32*.

**Table 4:** Infection of mice with *B. burgdorferi* clones lacking or containing *bbk17*.^a^

		Reisolation from tissues^c^				
				
Clone	Serology^b^	Ear	Bladder	Joint	No. of mice infected/total	*P*-value^d^
A3-M9 lp36-minus/pBSV2G	1/15	1/15	0/15	0/15	1/15	< 0.0001
A3-M9 lp36-minus/pBSV2G *bbk17*	15/15	15/15	15/15	15/15	15/15	
A3-M9 Δ*bbk17*::*flgB*_*p*_*-kan*/pBSV2G	1/15	0/15	0/15	1/15	1/15	< 0.0001
A3-M9 Δ*bbk17*::*flgB*_*p*_*-kan*/pBSV2G *bbk17*	13/15	13/15	13/15	13/15	13/15	

a Mice were infected by needle inoculation with 5 × 10^3^ spirochetes as described in the *Experimental procedures*. Data shown are pooled from three separate infection experiments.

b Assessed by immunoblot analysis with cell lysates of *B. burgdorferi* and *E. coli* producing P39 recombinant protein at 3 and 6 weeks post inoculation.

c Mice were sacrificed and tissues harvested 6 weeks post inoculation.

d The *P*-value was calculated by Fischer's exact test for the number of infected mice in the two groups.

### Inactivation of *bbk17* attenuates *B. burgdorferi* infection in mice

Because addition of the *bbk17* gene alone restored infectivity to spirochetes lacking lp36, this gene should be required for the ability of *B. burgdorferi* to infect the mammalian host. In order to test this hypothesis, we deleted the *bbk17* gene from the wild-type infectious clone A3-M9 and transformed the mutant with either the empty shuttle vector, pBSV2G (Elias *et al*., 2003) or with the shuttle vector containing *bbk17*, pBSV2G *bbk17*. Deletion of the *bbk17* gene had no effect on spirochetal growth *in vitro* (data not shown). The infectious phenotype of *B. burgdorferi* lacking only the *bbk17* gene (Δ*bbk17*) was assessed by a combination of intraperitoneal and subcutaneous needle inoculation of a target dose of 5 × 10^3^ spirochetes into a total of 15 C3H/HeN mice per clone in three experiments. Serological analysis of mice infected with Δ*bbk17* spirochetes demonstrated a weak antibody response limited to the *Borrelia* antigen P39 (Simpson *et al*., 1991) in only one out of the 15 mice inoculated (Table 4 and data not shown). Reisolation of Δ*bbk17* spirochetes was limited to the joint tissue of the single weakly seropositive mouse (Table 4). In contrast, the isogenic clone complemented with the *bbk17* gene on pBSV2G (*bbk17*^+^) elicited strong seroreactivity towards all *Borrelia* antigens in 13 out of the 15 mice inoculated, and *bbk17*^+^ spirochetes were reisolated from all three tissue sources (Table 4). These data suggest that the *bbk17* gene is a component of lp36 that contributes significantly to the critical role of this plasmid in mouse infectivity.

Analysis of the 50% infectious dose of Δ*bbk17* and *bbk17*^+^ spirochetes demonstrated a statistically significant right shift (*P* = 0.015) in the dose–response curve for the Δ*bbk17* clone relative to its isogenic complement (Fig. 4), indicating a *bbk17*-dependent significant difference in mouse infectivity. However, this only represented an approximate 10-fold increase in the ID_50_ for *B. burgdorferi* lacking the *bbk17* gene compared with the complemented clone, which were estimated to be 8.5 × 10^3^ (95% CI: 1.8 × 10^3^ − 4.6 × 10^4^) and 6.6 × 10^2^ (95% CI: 9.3 × 10^1^ − 3.3 × 10^3^) spirochetes, respectively (Table S1), and not the 4-log increase in ID_50_ observed for spirochetes lacking the entire lp36 plasmid. Furthermore, no differences in spirochetal loads in ear and heart tissues were detected between infected mice inoculated with 1 × 10^4^Δ*bbk17* or *bbk17*^+^ spirochetes (Fig. 3D, *P* > 0.05), suggesting that once mice become infected with either of these clones, there is no *bbk17*-dependent difference in spirochete burden among tissues. Although deletion of *bbk17* resulted in an increase in ID_50_ compared with the isogenic complement, additional genes on lp36 may be required for mouse infectivity. Together these data suggest that the *bbk17* gene is one of several genes on lp36 that contribute, in a dose-dependent manner, to mouse infection by *B. burgdorferi*. This result was unanticipated given the previous observation that addition of *bbk17* alone to spirochetes lacking the entire lp36 plasmid restored mouse infectivity.

### The *bbk17* gene encodes an adenine deaminase

The *bbk17* gene encodes a 548 amino acid protein that shares 33% and 31% amino acid identity with the adenine deaminase enzymes (AdeC) from *Bacillus subtilis* and *Escherichia coli*, respectively (Nygaard *et al*., 1996; Matsui *et al*., 2001). BBK17 is a member of the aminohydrolase super family of metal-dependent hydrolases, pfam01979 (Marchler-Bauer *et al*., 2005) and harbours two conserved domains, cd01295 and COG1001, characteristic of adenine deaminase enzymes (Marchler-Bauer *et al*., 2005).

**Fig. 5:**
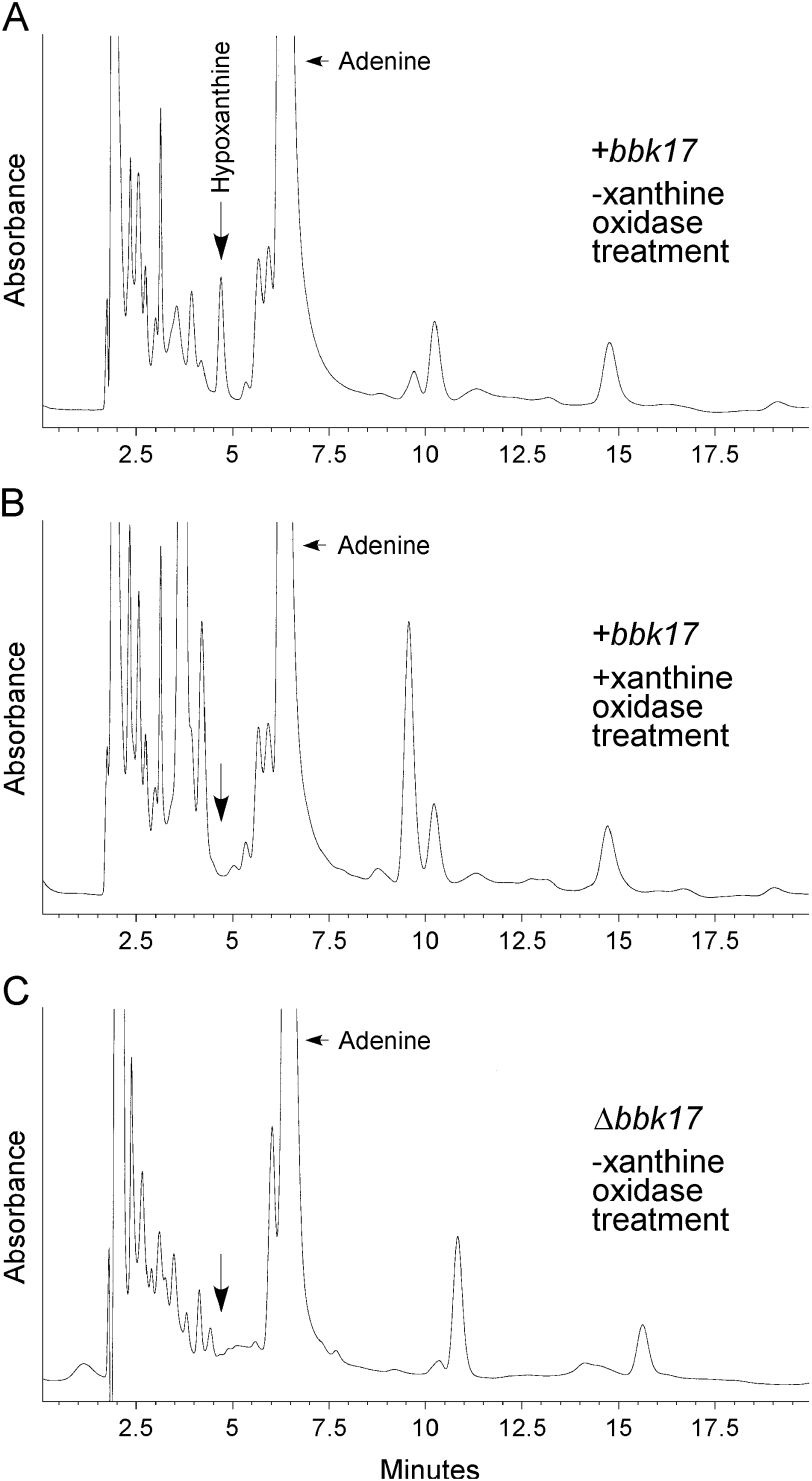
HPLC chromatograms of adenine deaminase enzyme assays from *B. burgdorferi* cell lysates containing or lacking *bbk17* A. A representative chromatogram for a cell lysate of wild-type clone A3-M9 30 min after the addition of 0.3 mM adenine (+*bbk17*, –xanthine oxidase treatment). Excess adenine was detected as a peak at a retention time of 6.3 min, identical to that of the adenine standard (data not shown). Hypoxanthine production was detected as a peak at a retention time of 4.7 min, identical to that of the hypoxanthine standard (data not shown). Peak retention times are shown in minutes along the *x*-axis. B. No peak at a retention time consistent with hypoxanthine was detected in an identical A3-M9 cell lysate sample 30 min after the addition of 0.3 mM adenine treated with xanthine oxidase (+*bbk17*, +xanthine oxidase treatment), as indicated by an arrow. C. A representative chromatogram for a cell lysate of clone A3-M9 Δ*bbk17*::*flg*_*p*_*-kan*/pBSV2G 30 min after the addition of 0.3 mM adenine (Δ*bbk17*, –xanthine oxidase treatment). No peak at a retention time consistent with hypoxanthine was detected in the sample lacking the *bbk17* gene, as indicated by an arrow.

Adenine deaminase (EC 3.5.4.2) is required for the direct deamination of adenine to produce hypoxanthine and is important for the salvage and metabolism of adenine in many prokaryotic species (Nygaard *et al*., 1996). The putative enzymatic function of the *bbk17* gene product was examined by quantifying the rate of hypoxanthine production from adenine over 30 min, using equivalent amounts of cell lysates made from *B. burgdorferi* clones containing or lacking the *bbk17* gene. All four *B. burgdorferi* clones harbouring the *bbk17* gene (wild-type A3-M9, lp36-minus/lp36-gent, lp36-minus/pBSV2G *bbk17* and Δ*bbk17*::*flgB*_p_-*kan*/pBSV2G *bbk17*) demonstrated adenine deaminase activity as measured by an HPLC assay for hypoxanthine production (Fig. 5, Table 5). A peak corresponding to the retention time of hypoxanthine was detected in the *bbk17*^+^ lysates using the reaction conditions described in the *Experimental procedures* (Fig. 5A). The identity of the hypoxanthine product was confirmed by the absence of this peak in equivalent samples treated with xanthine oxidase (EC 1.1.3.22) (Fig. 5B), an enzyme that degrades hypoxanthine to xanthine and finally to uric acid. No increase in hypoxanthine production was detected over the 30 min reaction time in the assay of cell lysates lacking the *bbk17* gene (lp36-minus, lp36-minus/pBSV2G and Δ*bbk17*::*flgB*_p_-*kan*/pBSV2G) (Fig. 5C, Table 5), indicating that the enzymatic activity necessary for conversion of adenine to hypoxanthine requires the *bbk17* gene product. Taken together, these data demonstrate that the *bbk17* gene encodes a protein with adenine deaminase activity and is appropriately annotated in the *B. burgdorferi* genome as *adeC* (Fraser *et al*., 1997; Casjens *et al*., 2000).

**Table 5:** Adenine deaminase activity of *B. burgdorferi* clones.

Clone	Adenine deaminase activity^a^ (pmol min^−1^ mg^−1^ total cell lysate ± SD)
A3-M9	17 ± 2.9
A3-M9 lp36-minus	−0.4 ± 0.7^b^
A3-M9 lp36-minus/lp36-gent	19 ± 0.2
A3-M9 lp36-minus/pBSV2G	−0.1 ± 0.6^b^
A3-M9 lp36-minus/pBSV2G *bbk17*	8.5 ± 0.2
A3-M9 Δ*bbk17*::*flgB*_*p*_*-kan*/pBSV2G	−0.2 ± 0.5^b^
A3-M9 Δ*bbk17*::*flgB*_p_-*kan*/pBSV2G *bbk17*	15 ± 2.4

a Adenine deaminase activity was determined by the rate of hypoxanthine produced over a 30 min period and expressed as the average change in hypoxanthine per minute per milligram of total protein in the cell lysate. Data represent the average of three experiments. Error is show as the standard deviation (SD) from the mean.

b No adenine deaminase activity was detected. A negative rate of hypoxanthine production was observed as a result of consumption of background levels of hypoxanthine by unidentified enzymatic activities present in the cell lysates.

## Discussion

In some cases *B. burgdorferi* plasmid loss has been shown to correlate with *in vivo* attenuation while having no effect on *in vitro* phenotypes. It is well established that linear plasmids lp25 and lp28-1 are required for *B. burgdorferi* survival in the mouse and/or the tick (Schwan *et al*., 1988a; Norris *et al*., 1995; Xu *et al*., 1996; 2005; Purser and Norris, 2000; Labandeira-Rey and Skare, 2001; McDowell *et al*., 2001; Labandeira-Rey *et al*., 2003; Grimm *et al*., 2004; 2005; Lawrenz *et al*., 2004; Strother *et al*., 2005). We have demonstrated that yet another linear plasmid, lp36, is critical for *B. burgdorferi* mouse infectivity.

### The lp36 plasmid is critical for *B. burgdorferi* mouse infectivity

*In vivo* phenotypic comparison between a strain B31-derived *B. burgdorferi* clone lacking lp36 and an isogenic clone harbouring the lp36 plasmid demonstrated that lp36-minus spirochetes are attenuated for maximal mouse infectivity when administered by needle inoculation, as well as by *Ixodes* larval and nymphal tick bite (Tables 2 and 3, Fig. 1), but have no defect at any stage of *Ixodes* tick infection (Fig. 2). A high inoculum of lp36-minus *B. burgdorferi* (> 10^6^ spirochetes) was required for consistent mouse infection by needle inoculation (Table S1). The number of spirochetes transmitted to mice by infected ticks is undefined, but some of the mice fed on by ticks carrying lp36-minus *B. burgdorferi* became infected (Table 3). The ability of tick transmitted spirochetes to infect a mouse may depend on the presence of tick-specific factors found in the saliva of the tick (Ribeiro, 1987; Ribeiro *et al*., 1987; 1990; Das *et al*., 2001; Zeidner *et al*., 2002; Montgomery *et al*., 2004; Ramamoorthi *et al*., 2005). In addition to the size of the inoculum and tick salivary components, the phenotypic state of *B. burgdorferi* transmitted from infected ticks relative to that of needle-injected, *in vitro* grown spirochetes (Schwan *et al*., 1995; Carroll *et al*., 1999; Ohnishi *et al*., 2001; Revel *et al*., 2002; Ojaimi *et al*., 2003; Ramamoorthi *et al*., 2005) could also contribute to the infectious capability of *Borrelia*.

The tissue source of the lp36-minus spirochetes infrequently reisolated from infected mice was mainly limited to the mouse ear (Table 3, Table S1). In addition, quantitative assessment of spirochetes in tissues of lp36-minus infected mice indicated that spirochetes were limited to ear tissue (Fig. 3A and B). Reduced reisolation and detection by qPCR of lp36-minus spirochetes from deeper mouse tissues, such as the bladder, joint and heart, may suggest that these spirochetes are less able to survive in those tissue microenvironments or that the *Borreliae* were less able to disseminate to these sites. Despite weak infection of a subset of mice fed on by ticks infected with lp36-minus spirochetes, uninfected ticks that fed on these mice did not acquire spirochetes, indicating that *B. burgdorferi* lacking lp36 would not be effectively maintained in the infectious cycle. Furthermore, the ID_50_ value for lp36-minus spirochetes was increased by almost 4-log relative to lp36^+^ bacteria (Table S1). This level of attenuation is similar to that of *B. burgdorferi* clones lacking the essential virulence plasmids lp25 and lp28-1, the ID_50_ values for which have been determined to be greater than 10^7^ and 10^5^ spirochetes, respectively (Labandeira-Rey and Skare, 2001; Purser *et al*., 2003). Together these data indicate that like lp25 and lp28-1, lp36 is vital for *B. burgdorferi* survival and persistence in the mammalian environment.

### The *bbk17* gene encodes an adenine deaminase and is important for *B. burgdorferi* survival in the mammalian environment

BBK17 is appropriately annotated in the *B. burgdorferi* genome as an adenine deaminase (AdeC) (Fraser *et al*., 1997; Casjens *et al*., 2000). Enzymatic conversion of adenine to hypoxanthine was detected in wild-type *B. burgdorferi* protein lysates (Fig. 5, Table 5). This activity could be attributed to the *bbk17* gene product because deletion of the entire lp36 plasmid, as well as deletion of the *bbk17* gene alone, resulted in loss of hypoxanthine production (Fig. 5, Table 5). Moreover, reconstitution of lp36 and complementation of the *bbk17* defect restored adenine deaminase activity (Table 5). *B. burgdorferi* does not harbour the genes encoding enzymes known to be required for *de novo* nucleotide/nucleoside biosynthesis and therefore presumably scavenges purines and pyrimadines from the host environment (Fraser *et al*., 1997; Casjens *et al*., 2000). Because of the putative biochemical function of the *bbk17* gene product, mutant clones lacking this gene were predicted to be auxotrophs in environments low in adenine and hypoxanthine. Spirochetes lacking the lp36 plasmid or the *bbk17* gene alone demonstrated no defect in growth *in vitro* (data not shown). However, BSK II is a complex, rich medium containing significant levels of free nucleotides, nucleosides and bases available for direct transport that may bypass *Borrelia*'s requirement for conversion of adenine to hypoxanthine. Similarly, the high concentration of hypoxanthine in blood (Hartwick *et al*., 1979), along with the availability of free nucleotides from lysed cells in the blood meal, may bypass the requirement for adenine de-aminase activity for survival of spirochetes in the tick midgut. While *B. burgdorferi* may be able to use the hypoxanthine in blood directly, in lieu of conversion of adenine to hypoxanthine, these spirochetes are only transiently present in the mammalian blood stream during dissemination to various tissues and do not replicate to high numbers in this microenvironment (Barthold *et al*., 1992; Wormser, 2006). It is in the tissues that *B. burgdorferi* replicates and causes a persistent infection in the mouse (Schwan *et al*., 1988b). Hypoxanthine appears to have a limited tissue distribution relative to adenine, which appears to be ubiquitous among mammalian tissues (Wishart *et al*., 2007). Therefore, the mammalian environment may present more of a challenge to *B. burgdorferi* for survival and may require AdeC for purine scavenge and biosynthesis. Consistent with this hypothesis, mutations in purine biosynthetic genes of various pathogenic bacterial species have resulted in a loss of virulence in animal models (Cersini *et al*., 2003; Alcantara *et al*., 2004; Pechous *et al*., 2006; Pilatz *et al*., 2006).

The mammalian infectivity defect of *B. burgdorferi* lacking the lp36 plasmid was indeed localized, in part, to the *bbk17* gene. Reintroduction of this gene alone under the control of its own promoter eliminated the lp36-dependent infectivity defect, although reduced spirochetal loads were detected in the heart tissue of infected mice compared that of mice infected with spirochetes harbouring the entire lp36 plasmid. These data suggest that in the absence of all other lp36 genes, addition of *bbk17* is sufficient for survival and persistence of *B. burgdorferi* in the mouse, although additional genes on lp36, such as *bbk32*, may be important to achieve maximum spirochetal loads in all tissues.

*In vivo* analysis of *B. burgdorferi* lacking just the *bbk17* gene at the target dose of 5 × 10^3^ spirochetes resulted in infection of only one out of 15 mice (Table 4). This attenuation was due to the absence of the *bbk17* gene, as complementation resulted in infection of 13 out of 15 mice (Table 4). ID_50_ analysis comparing Δ*bbk17* and *bbk17*^+^ spirochetes only resulted in an approximate 10-fold difference in the ID_50_ values (Fig. 4, Table S1). These results were unexpected given the striking attenuation of spirochetes lacking *bbk17* at a dose of 5 × 10^3^ spirochetes, as well as the ability of the *bbk17* gene alone to restore mouse infectivity to spirochetes lacking the entire lp36 plasmid. The adenine deaminase activity of the *bbk17* gene may provide a survival advantage for *B. burgdorferi* in this infection model; however, additional genes present on lp36 appear to also contribute to *B. burgdorferi* colonization and survival within the mouse. Furthermore, the role of *bbk17* in mouse infectivity appears to be affected significantly by relatively small differences in the dose of the inoculum. Similarly, deletion of the *bbk32* gene, also encoded on lp36, results in an approximate 10-fold increase in ID_50_ relative to the complemented strain (1.8 × 10^3^ and 80 spirochetes respectively) (Seshu *et al*., 2006); whereas Δ*bbk32 B. burgdorferi* established infections in all mice inoculated with 1 × 10^5^ spirochetes (Li *et al*., 2006). These data suggest that both the *bbk17* and *bbk32* genes, perhaps along with additional lp36 genes, are important for colonization, dissemination and persistence of *B. burgdorferi* in the mammalian environment.

## Conclusion

Functions encoded by lp36 are critical for *B. burgdorferi* mammalian infectivity. The importance of lp36 provides additional support to the conclusion that genes vital to the survival of *B. burgdorferi in vivo* can be located on plasmids (Schwan *et al*., 1988a; Norris *et al*., 1995; Xu *et al*., 1996; 2005; Purser and Norris, 2000; Labandeira-Rey and Skare, 2001; McDowell *et al*., 2001; Labandeira-Rey *et al*., 2003; Byram *et al*., 2004; Grimm *et al*., 2004; 2005; Lawrenz *et al*., 2004; Strother *et al*., 2005). This is an unusual feature of the *Borrelia* genome, as plasmids of other bacterial species typically carry non-essential genes that confer a selective advantage in a particular environment. The *bbk17* gene has been identified as a component of lp36 that contributes to the essential nature of this plasmid by fulfilling a biochemical function important for *B. burgdorferi* survival, thereby providing greater insight into the genetic basis for the complex lifestyle of *B. burgdorferi*.

## Experimental procedures

### *Borrelia burgdorferi* clones and growth conditions

All low-passage infectious *B. burgdorferi* clones used in this study are listed in Table 1 and are derived from strain B31 clone A3, which lacks the plasmid cp9 but contains all 20 additional plasmids described in the parental strain MI-B31 (Elias *et al*., 2002). Wild-type clone A3-M9 was derived by passage of A3 through a mouse, followed by single colony isolation (Tilly *et al*., 2004). A3-M9 lacks both cp9 and lp21, which have been demonstrated to be dispensable for mouse and tick infection (Purser and Norris, 2000; Elias *et al*., 2002; Tilly *et al*., 2004). A3-M9 lp36-minus is a derivative of the parent clone A3-M9 that lost lp36 during outgrowth from a frozen stock. *B. burgdorferi* were grown in liquid Barbour-Stoenner-Kelly (BSK) II medium supplemented with gelatin and 6% rabbit serum (Barbour, 1984) and plated in solid BSK medium as previously described (Rosa and Hogan, 1992; Samuels, 1995). All cultures were grown at 35°C with 2.5% CO_2_. Kanamycin was used at 200 μg ml^−1^ and gentamicin at 40 μg ml^−1^.

### Construction and restoration of lp36-gent

Plasmid lp36-gent was constructed by allelic exchange with a 1.8 kb fragment of lp36 encompassing the intergenic region between ORFs K04 and K05 (position 3590–5424) harbouring the *flaB*_p_-*aacC1* gentamicin resistance cassette inserted into the BamHI site at position 4554 on lp36. All restriction enzymes were purchased from New England Biolabs. Use of the BamHI site on lp36 required the removal of the BamHI site from the cloning vector pOK12 (Vieira and Messing, 1991) by restriction digestion of pOK12 with EcoRV and StuI, followed by religation. The 1.8 kb fragment spanning nucleotides 3590–5424 of lp36 was amplified from B31 clone A3 genomic DNA using primers 1 and 2 (Table S2) and cloned into pGEM-T easy (Promega). The lp36 DNA fragment was removed from pGEM-T easy by restriction digestion with NotI. The resulting DNA fragment was ligated into NotI-digested pOK12 lacking BamHI. The *flaB*_p_-*aacC1* gentamicin resistance cassette (Elias *et al*., 2003) was amplified using primers 3 and 4 (Table S2) and cloned into pCR2.1-TOPO (Invitrogen). The gentamicin resistance cassette was removed from pCR2.1-TOPO by restriction digestion with BamHI and ligated into the BamHI site within the 1.8 kb DNA fragment of lp36 cloned into pOK12 lacking BamHI. The structure of pOK12-lp36-gent was confirmed by sequence analysis. 20 μg of pOK12-lp36-gent plasmid DNA purified from *E. coli* was transformed into B31 clone A3 as previously described (Samuels, 1995; Elias *et al*., 2002; Grimm *et al*., 2004) and transformants selected on solid BSK medium containing gentamicin. Colonies were screened by PCR for the presence of the gentamicin cassette on lp36 using primers 1 and 2 (Table S2). To restore lp36 to clone A3-M9 lp36-minus, total plasmid DNA was purified from A3/lp36-gent and transformed into A3-M9 lp36-minus. Gentamicin-resistant colonies were screened by PCR for the presence of the antibiotic marker on lp36 using primers 1 and 2 (Table S2). Total genomic DNA was prepared from lp36-gent PCR positive clones and screened with a panel of primers for the presence of all *B. burgdorferi* plasmids (Elias *et al*., 2002). A clone that retained the *B. burgdorferi* plasmid content of the parent clone was used in further experiments (Table 1). Restoration of lp36 was further confirmed by Southern hybridization (data not shown).

### Deletion of *bbk17*

The *bbk17* gene was deleted from lp36 by allelic exchange with the kanamycin resistance cassette, *flgB*_p_-*kan* (Bono *et al*., 2000). A 2.9 kb fragment including *bbk17*, 600 bp of upstream sequence and 500 bp of downstream sequence was amplified from B31 clone A3 genomic DNA using the Expand Long PCR system (Roche) and primers 5 and 6 (Table S2) and cloned into pCR-XL TOPO (Invitrogen), resulting in p*bbk17*. The 1.8 kb *bbk17* DNA fragment was removed from p*bbk17* by inverse PCR using the Expand Long PCR system (Roche) and primers 7 and 8 (Table S2), yielding linear pΔ*bbk17* with SalI sites at its ends. The kanamycin resistance cassette, *flgB*_p_-*kan* (Bono *et al*., 2000), was amplified from pBSV2 (Stewart *et al*., 2001) with XhoI ends using primers 9 and 10 (Table S2). The *flgB*_p_-*kan* cassette was digested with XhoI and ligated into SalI cut-pΔ*bbk17*, yielding pΔ*bbk17*::*kan*. 20 μg of pΔ*bbk17*::*kan* plasmid DNA purified from *E. coli* was transformed into A3-M9 as previously described (Samuels, 1995; Elias *et al*., 2002; Grimm *et al*., 2004) and the recombinants were selected in solid BSK medium containing kanamycin. Colonies were screened by PCR for the presence of the kanamycin resistance cassette in place of the *bbk17* gene using primers 7 and 8 (Table S2). Total genomic DNA was prepared from PCR-positive A3-M9 Δ*bbk17*::*flgB*_p_-*kan* clones and screened with a panel of primers for the presence of all *B. burgdorferi* plasmids (Elias *et al*., 2002). A clone that retained the *B. burgdorferi* plasmid content of the parent clone was used in further experiments (Table 1).

### Construction of *pBSV2G bbk17*

The *bbk17::flgB*_p_-*kan* mutant was complemented with pBSV2G *bbk17*, a shuttle vector carrying a wild-type copy of the *bbk17* gene and its own promoter. This plasmid was also used to reintroduce *bbk17* alone to the A3-M9 lp36-minus clone. Plasmid pBSV2G *bbk17* was constructed by PCR-amplifying a 1.8 kb DNA fragment containing the *bbk17* gene and its putative promoter region from strain B31 clone A3 using Vent polymerase (Invitrogen) and primers 11 and 12 (Table S2). The *bbk17* gene was digested with KpnI and BamHI and directionally cloned into the *B. burgdorferi* shuttle vector pBSV2G (Elias *et al*., 2003) digested with KpnI and BamHI. Plasmid structure and wild-type sequence were analysed and verified by PCR with primers 11 and 12 (Table S2), restriction digestion and sequence analysis. To facilitate transformation of pBSV2G *bkk17* into the low passage infectious clone, the *E. coli* purified plasmid was first transformed into the high passage, non-infectious clone, B31-A (Byram *et al*., 2004). B31-A lacks lp25 but retains lp56, both of which carry restriction modification systems (Lawrenz *et al*., 2002; Kawabata *et al*., 2004). Purification of pBSV2G *bbk17* from lp56^+^ B31-A should result in partially modified *B. burgdorferi* DNA that may be less likely to be targeted for degradation upon transformation into the lp25^+^, lp56^+^ low passage infectious clone. *B. burgdorferi* clones A3-M9 Δ*bbk17*::*flgB*_*p*_*-kan* and A3-M9 lp36-minus were transformed with pBSV2G *bbk17* purified from B31-A or pBSV2G alone purified from *E. coli* (Samuels, 1995; Elias *et al*., 2002; Grimm *et al*., 2004). Transformants were screened by PCR for the presence of the *bbk17* and *aacC1* genes, or the *aacC1* gene alone using primer pairs 11 and 12 and 4 and 9 (Table S2). PCR-positive transformants were analysed for plasmid content and clones that retained the plasmids found in the parent clone were selected for further experiments (Table 1).

### In vitro growth analysis

*Borrelia burgdorferi* clones were inoculated from frozen stocks into 5 ml of BSK II medium containing the appropriate antibiotic and grown to an approximate density of 1 × 10^7^ spirochetes ml^−1^. Clones were subsequently diluted in triplicate to 1 × 10^5^ spirochetes ml^−1^ in 5 ml of BSK II containing the appropriate antibiotic. Spirochete density was determined every 24 h over 120–140 h using a Petroff-Hausser counting chamber.

### Experimental tick-mouse infectious cycle

The Rocky Mountain Laboratories (RML) is accredited by the International Association for Assessment and Accreditation of Laboratory Animal Care. Protocols for all animal experiments were prepared according to the guidelines of the National Institutes of Health and approved by the RML's Animal Care and Use Committee. Two different strains of mice were used in these experiments. Mice from an outbred colony of Swiss-Webster mice maintained at RML represent a genetically heterogeneous rodent population (called RML mice) and C3H/HeN mice (Harlan Sprague-Dawley, Indianapolis, IN) represent a uniform, inbred rodent population. Needle inoculations of mice were performed by 80% intraperitoneal and 20% subcutaneous inoculation with a target dose of 5 × 10^3^ spirochetes per mouse, using three to nine mice per *B. burgdorferi* strain, and were performed at least twice. The number of spirochetes inoculated into mice was determined using a Petroff-Hausser counting chamber and verified by colony-forming unit (cfu) counts in solid BSK medium; 10–20 colonies per inoculum were screened by PCR for the presence of the virulence plasmids lp25 and lp28-1. Actual doses were calculated by adjusting the target doses for the observed spirochete densities determined from the colony counts and percentages of spirochetes carrying lp25 and lp28-1. Total plasmid contents of all *in vivo* inoculum cultures were verified using a panel of primers that amplify specific DNA targets on all *B. burgdorferi* plasmids (Elias *et al*., 2002). Mouse infection was assessed 3–7 weeks post inoculation by immunoblot analysis of mouse sera and reisolation of spirochetes from ear, bladder and joint tissues, as previously described (Simpson *et al*., 1991; Elias *et al*., 2002; Grimm *et al*., 2003; 2004). Fischer's exact test was used to generate a *P*-value for the test of no difference between the numbers of mice infected with different *B. burgdorferi* clones. The analysis was implemented using R software, version 2.2.1 (R Development Core Team, 2005).

Cohorts of 100–200 4-month-old *I. scapularis* tick larvae (from a colony maintained at RML, Hamilton, MT) were experimentally infected with equal density, exponential phase cultures of various *B. burgdorferi* clones as previously described (Policastro and Schwan, 2003; Grimm *et al*., 2005). Each immersion was performed in duplicate and the entire experiment performed at least twice. Infection of larvae and nymphs (following the molt) were assessed 7–14 days post feeding to repletion (100–200 larvae or 15–25 nymphs were applied per mouse) by IFA (Schwan and Piesman, 2000) and spirochete density per tick quantified by plating of dilutions of triturated whole ticks in solid BSK medium, as previously described (Grimm *et al*., 2005). Infection of RML mice fed on by infected ticks was assessed 3–9 weeks post tick feeding by seroconversion to *B. burgdorferi* antigens (Simpson *et al*., 1991; Elias *et al*., 2002; Grimm *et al*., 2003; 2004), reisolation of spirochetes from mouse tissues (Elias *et al*., 2002; Grimm *et al*., 2004) and xenodiagnosis using uninfected *I. scapularis* (RML) (Schwan and Piesman, 2000). Fischer's exact test was used to generate a *P*-value for the test of no difference between the numbers of mice infected with different *B. burgdorferi* clones. The analysis was implemented using R software, version 2.2.1 (R Development Core Team, 2005).

### Determination of ID_50_

The dose required to infect half the of the mice inoculated (ID_50_) was experimentally determined for *B. burgdorferi* clones A3-M9 lp36-minus, A3-M9 lp36-minus/lp36-gent, A3-M9 lp36-minus/pBSV2G *bbk17*, A3-M9 Δ*bbk17*::*flgB*_p_-*kan*/pBSV2G and A3-M9 Δ*bbk17*::*flgB*_p_-*kan*/pBSV2G *bbk17*. Groups of 2–6 C3H/HeN mice (Harlan Sprague-Dawley, Indianapolis, IN) were inoculated with 10-fold increasing doses of spirochetes estimated to flank the ID_50_, as described above. Target dose ranges for *B. burgdorferi* clones were as follows: A3-M9 lp36-minus: 1 × 10^4^ − 1 × 10^8^; A3-M9 lp36-minus/lp36-gent: 1 × 10^1^ − 1 × 10^8^; A3-M9 lp36-minus/pBSV2G *bbk17*: 1 × 10^2^ − 1 × 10^6^; A3-M9 Δ*bbk17*::*flgB*_p_-*kan*/pBSV2G: 1 × 10^3^ − 1 × 10^7^ and A3-M9 Δ*bbk17*::*flgB*_p_-*kan*/pBSV2G *bbk17*: 1 × 10^2^ − 1 × 10^5^. Actual doses were calculated by adjusting the target doses for the observed spirochete densities determined from the colony counts and percentages of spirochetes carrying lp25 and lp28-1. Two mice were inoculated with BSK II medium alone as negative controls. Mouse infection was assessed 4-weeks post inoculation as described above.

The data from the ID_50_ infection experiment and the single dose infection experiment for each clone were combined for the estimations of the 50% infectious dose. The ID_50_ value for each clone was derived using probit regression with a separate location parameter (intercept) for each clone to fit clone-specific curves to the proportions of mice infected versus the log_10_(adjusted dose). This model assumed identical slopes for the dose–response curve of each clone. With this parameterization, the differences in infectivity between the different clones can be seen as lateral shifts of the dose–response curve along the dose axis (Fig. 4). The log_10_ID_50_ for each *Borrelia* clone was derived by finding the place on its fitted curve that corresponds to the 50% infection mark and then converting the log-dose value back to the original scale to give the ID_50_ (Table S1). The model also fitted an overdispersion factor that adjusts the standard errors and widens the confidence intervals otherwise derived from the model. This was needed to account for greater heterogeneity in the infection rates than explained by dose and *Borrelia* clone alone. The analysis was implemented using R software, version 2.2.1 (R Development Core Team, 2005).

### Quantitative assessment of spirochetal loads in mouse tissues

Ear, heart and joint tissues were harvested from inoculated mice at the time of sacrifice. Total mouse and spirochete DNA was isolated from tissues as previously described (Seiler *et al*., 1995) using collagenase A (Roche, Indianapolis, IN), proteinase K (Invitrogen), and DNase-free RNase (QIAGEN) digestions, in combination with phenol-chloroform and chloroform extractions and ethanol precipitations. DNA was resuspended in 100 μl of DNase/RNase-free water and run through a QIAquick PCR purification column (QIAGEN). The concentration of the purified DNA was measured at 260 nm and diluted to 50 μg ml^−1^ in DNase/RNase-free water. Real-time PCR to quantify *B. burgdorferi* genomes with respect to mouse genomes was performed with TaqMan primers and probes (Sigma-Genosys) (Table S2) for the *flaB* gene (*B. burgdorferi* chromosome) (primers 13 and 14, probe 1) and the mouse *nidogen* gene (Morrison *et al*., 1999) (primers 15 and 16, probe 2) using an Applied Biosystems 7900HT instrument. Samples were analysed in triplicate and the spirochete burden was expressed as *flaB* spirochete DNA copies per 1000 *nid* mouse DNA copies. Data sets were compared using one-way anova with Tukey's post test using GraphPad Prism version 4.00 for Windows (GraphPad Software, San Diego California USA).

### Adenine deaminase enzyme activity assay

*Borrelia burgdorferi* clones were grown in 250 ml of BSK II medium containing the appropriate antibiotic(s) to an approximate density of 7 × 10^7^ cells ml^−1^. Spirochetes were harvested by centrifugation and washed twice with 10 mM NaCl, 20 mM HEPES pH 7.6. Cell pellets were resuspended in 5 ml of 50 mM Tris pH 7.5 and lysed by French press (ThermoSpectronic) at 14 000 psi for three passes. Lysates were cleared by centifugation, aliquoted and stored at −20°C.

Five hundred microlitres of cell lysate were added to assay mixture (1 ml final) containing 40 mM Tris pH 7.5, 5 mM MnCl_2_ and 0.3 mM adenine. Reactions were incubated at 37°C for 30 min. Two hundred and thirty microlitres were removed from the reaction mixture, in duplicate, at time 0 and 30 min and heat inactivated at 95°C for 10 min. One set of duplicates was treated with 1 μl of (0.03 U) xanthine oxidase (EC 1.1.3.22, Sigma), incubated at 37°C for 30 min and heat inactivated as described above. All samples were cleared by centrifugation prior to HPLC analysis.

Hypoxanthine production was analysed using an Agilent 1100 Series HPLC system connected to a Supelco C18, 4.6 × 150 mm reverse phase column (Sigma) protected with a guard column of identical composition. One hundred microlitres of samples were injected via the auto-sampler onto the column equilibrated in and eluted isocratically with 500 mM potassium phosphate pH 4.0 and 2% methanol. Absorbance during elution was monitored at 254 nm for 20 min. Peak identity was determined by retention time as compared with authentic standards. In addition, the composition of the hypoxanthine peak was confirmed by absence of the peak following xanthine oxidase treatment. Peak area was determined by integration and quantified against a calibration curve prepared using 99% pure hypoxanthine (Sigma). Adenine deaminase activity was calculated as the rate of increase in hypoxanthine over 30 min. Protein concentrations were measured using the Bicinchoninic Acid Protein Assay Kit (Sigma). Adenine deaminase activity is presented as the picomoles of hypoxanthine produced per minute per milligram of total protein in each lysate. Assays were performed in triplicate and error is given as the standard deviation from the mean.

## Supplementary material

The following supplementary material is available for this article:

Table S1 Determination of ID_50_ of various *B. burgdorferi* clones. Groups of C3H/HeN mice were inoculated with 10-fold increasing target doses of spirochetes. Mouse infection was assessed 4-weeks post inoculation by reisolation of spirochetes from ear, bladder and joint tissues. The ID_50_ value for each clone was estimated using probit regression with a separate location parameter (intercept) for each clone to fit clone-specific curves to the proportion of mice infected versus the log_10_(adjusted dose) (Fig. 3). The log_10_ID_50_ for each *Borrelia* clone was derived by finding the place on its fitted curve that corresponded to the 50% infection mark and then converting the log-dose back to the original scale to give the ID_50_. 95% confidence intervals for each ID_50_ value are shown.

Table S2 Primers and TaqMan probes used in this study.

This material is available as part of the online article from:

http://www.blackwell-synergy.com/doi/abs/10.1111/j.1365-2958.2007.05746.x

(This link will take you to the article abstract).
