# Supplementary material for: The critical role of the linear plasmid lp36 in the infectious cycle of Borrelia burgdorferi
Source: Mol Microbiol. 2007 Jun 1;64(5):1358–74. doi: 10.1111/j.1365-2958.2007.05746.x (PMC1974800; doi:10.1111/j.1365-2958.2007.05746.x)
Supplement: Table S1 — Determination of ID50 of various B. burgdorferi clones. Groups of C3H/HeN mice were inoculated with 10-fold increasing target doses of spirochetes. Mouse infection was assessed 4-weeks post inoculation by reisolation of spirochetes from ear, bladder and joint tissues. The ID50 value for each clone was estimated using probit regression with a separate location parameter (intercept) for each clone to fit clone-specific curves to the proportion of mice infected versus the log10(adjusted dose) (Fig. 3). The log10ID50 for each Borrelia clone was derived by finding the place on its fitted curve that corresponded to the 50% infection mark and then converting the log-dose back to the original scale to give the ID50. 95% confidence intervals for each ID50 value are shown. [file mmi0064-1358-ts1.pdf]

Table S1. Determination of ID_{50}[a].

Target Dose	A3-M9 lp36-minus	A3-M9 lp36-minus/ lp36-gent	A3-M9 lp36-minus/ pBSV2G *bbk17*	A3-M9 Δ*bbk17*::*flgB*$_p$-*kan*/ pBSV2G	A3-M9 Δ*bbk17*::*flgB*$_p$-*kan*/ pBSV2G *bbk17*
$1x10^1$	ND[b]	0/6	ND	ND	ND
$1x10^2$	ND	1/6	1/6	ND	0/6
$1x10^3$	ND	3/6	3/6	1/6	3/6
$5x10^3$ [c]	1[d] /15	14/15	15/15	1/15	13/15
$1x10^4$	0/6	2/2	4/6	5/6	6/6
$1x10^5$	1[d]/6	2/2	6/6	6/6	6/6
$1x10^6$	0/6	2/2	6/6	6/6	ND
$1x10^7$	3[d]/6	2/2	ND	6/6	ND
$1x10^8$	4[d]/6	2/2	ND	ND	ND
ID_{50}[e]	$7.1x10^6$	$9.5x10^2$	$4.1\ x10^2$	$8.5\ x10^3$	$6.6\ x10^2$
95% CI[f]	$1.1x10^6$-$5.8x10^7$	$1.5x10^2$-$5.0x10^3$	$5.5x10^1$-$2.1x10^3$	$1.8x10^3$-$4.6x10^4$	$9.3x10^1$-$3.3x10^3$

[a]Number of mice infected assessed by reisolation of spirochetes from mouse ear, bladder and joint tissues 4 weeks post inoculation/number of mice analyzed. Spirochetes were reisolated from all three tissues unless otherwise noted.

[b]Not Determined.

[c]Includes infection data from a separate experiment in which mice were inoculated with a single target dose of $5x10^3$.

[d]Spirochetes were only reisolated from ear tissue.

[e]Estimated by probit regression on adjusted dose as described in the Experimental Procedures.

[f]95% Confidence Interval.

Table S2. Primers and Taqman probes used in this study.

Primer number	Designation	Sequence 5′-3′
1	lp36 F	CGTCAAATCTTCTGGGGTG
2	lp36 R	CCTATTTCAAGGGCGTGAG
3	*flaB*$_p$-BamHI	GGATCCTGTCTGTCGCCTCTTGTGGCTTCCGG
4	3′ *aacC1*-NheI	GCTAGCCGATCTCGGCTTGAACG
5	*bbk17* –600 5′	ATGCAAAATGCGGGCATAGAATTCC
6	*bbk17* +500 3′	AAGGATGCTATTAAAAGCGGAGCCG
7	*bbk17*-12045-SalI 3′	ACGCGTCGACGGATTATTAATTGTGTGTCTGGCAG
8	*bbk17*-10242-SalI 5′	ACGCGTCGACCTTAAATTATAGCGCAAAGTGCCAG
9	*flgB*P$_o$ XhoI	TAATACTCGAGCTTCAAGGAAGATTT
10	*Kan* term- XhoI	ATCTCGAGCTAGCGCCGTCCCGTCAA
11	*bbk17*-KpnI 5′	CGGGGTACCCTTTGCGCTATAATTTAAGT
12	*bbk17*-BamHI 3′	CGCGGATCCAAAAACTTTTGAGTTCCTTC
13	*flaB* FWD	TCTTTTCTCTGGTGAGGGAGCT
14	*flaB* REV	TCCTTCCTGTTGAACACCCTCT

15	*nid* FWD	CACCCAGCTTCGGCTCAGTA
16	*nid* REV	TCCCCAGGCCATCGGT

Probe number	Designation	Sequence 5′-3′
1	*flaB* Taqman probe	6-FAM-AAACTGCTCAGGCTGCACCGGTTC-TAMRA
2	*nid* Taqman probe	6-FAM-CGCCTTTCCTGGCTGACTTGGACA-TAMRA
